# Impact of baseline lymphopenia on prognosis of patients with septic shock

**DOI:** 10.1186/s12879-025-12078-9

**Published:** 2025-12-15

**Authors:** Yuan-Yuan Li, Yan Chen, Shan Li, Wei Jiang, Xiao-Yun Hu, Chun-Yao Wang, Run Dong, Li Weng, Jin-Min Peng, Bin Du

**Affiliations:** 1 Medical ICU, Peking Union Medical College Hospital, Peking Union Medical College and Chinese Academy of Medical Sciences, No. 1 Shuai Fu Yuan, Beijing, 100730 China; 2 Department of Critical Care Medicine, Shandong Provincial Hospital Affiliated to Shandong First Medical University, Jinan, 250021 China

**Keywords:** Septic shock, Lymphopenia, Mortality

## Abstract

**Background:**

Sepsis is defined as life-threatening organ dysfunction due to a dysregulated host response to infection. Lymphocytes play an important role in sepsis, but little is known about the comparisons of different lymphopenia and non-lymphopenia patients with septic shock. The aim of this study is to compare the clinical characteristics of patients with septic shock among pre-existing lymphopenia group, sepsis-induced lymphopenia group, and non-lymphopenia group, and to identify the relationship of different lymphopenia with outcomes.

**Methods:**

This retrospective study included 354 septic-shock patients with baseline lymphocyte available during 2013 and 2023, which were divided into pre-existing lymphopenia group, sepsis-induced lymphopenia group, and non-lymphopenia group. We compared the clinical characteristics and identified the relationship of different lymphopenia with 30-day mortality by univariate analysis and multivariable logistic regression analysis.

**Results:**

Among 354 patients with septic shock with baseline lymphocyte available, the pre-existing lymphopenia group (*n* = 159) exhibited the highest 30-day mortality (50.9%), while the mortality in sepsis-induced lymphopenia patients (*n* = 143) and non-lymphopenia groups (*n* = 52) was 37.1% and 44.2%, respectively (*p* = 0.069). Multivariable analysis showed that pre-existing lymphopenia was an independent risk factor for 30-day mortality (OR 3.67, 95% CI 1.61–8.79), while lymphopenia at ICU admission was not (OR 0.95, 95% CI 0.28–3.37).

**Conclusion:**

Pre-existing lymphopenia was an independent risk factor for 30-day mortality in patients with septic shock, while sepsis-induced lymphopenia at ICU admission was not. These results suggest that historical lymphocyte count level should be incorporated into risk stratification paradigms for septic shock patients.

**Supplementary information:**

The online version contains supplementary material available at 10.1186/s12879-025-12078-9.

## Introduction

Sepsis is a life-threatening condition caused by a dysregulated host response to infection and remains a major cause of morbidity, mortality and global health burden [1]. Lymphocytes play a central role in the immune regulation of sepsis by orchestrating both innate and adaptive immune responses. Lymphopenia is common laboratory finding in patients with sepsis, primarily resulting from increased lymphocyte apoptosis and impaired lymphopoiesis [2–4].

Accumulating evidence indicates that lymphopenia is closely associated with immune dysfunction and poor clinical outcomes, including secondary infection, multiple organ failure, and increased mortality [5–7]. Moreover, both the timing and persistence of lymphopenia appear to be better predictors of prognosis, and lymphopenia has thus been proposed as a potential prognostic marker in patients with sepsis. However, most existing studies have focused on sepsis-induced lymphopenia, and patients with pre-existing lymphopenia were often excluded from clinical cohorts, leaving their prognostic significance insufficiently characterized. Pre-existing lymphopenia may result from various causes, including pharmacological interventions, underlying conditions such as malignancy or autoimmune disease [8]， [9], malnutrition [10], and chronic alcohol abuse [11], [12]. A large population-based cohort of 98,344 individuals revealed that lymphopenia was associated with a multivariable-adjusted hazard ratio of 1.70 for infection-related death [13], suggesting that pre-infection lymphopenia might reflect a predisposition to infection-related adverse outcomes. Nevertheless, it remains unclear whether pre-existing lymphopenia is associated with higher mortality in patients with septic shock, or whether these patients experience disease progression and outcomes similar to those who develop lymphopenia secondary to sepsis.

Therefore, this study aimed to compare the clinical characteristics and outcomes of septic shock patients with pre-existing lymphopenia, sepsis-induced lymphopenia, and normal lymphocyte counts, and to evaluate the association between pre-existing lymphopenia and 30-day mortality.

### Methods

This retrospective study enrolled patients with septic shock admitted to ICUs (general ICU, medical ICU, emergency ICU, and respiratory ICU) at Peking Union Medical College Hospital from January 1, 2013, to June 30, 2023. This study was approved by the Peking Union Medical College Hospital Ethics Committee (K5337) and was conducted in accordance with the 1964 Declaration of Helsinki and its later amendments. Informed consent was waived due to its retrospective nature.

This study included patients meeting the following inclusion criteria: (1) Adult patients diagnosed with septic shock according to the Sepsis-3 criteria [14]; (2) an ICU length of stay exceeding 48 hours; and (3) availability of peripheral blood lymphocyte count obtained within 7–30 days prior to ICU admission. Exclusion criteria: (1) cases without outcome data; (2) patients transferred from another hospital’s ICU; and (3) confirmed occurrence of another infection within one month before ICU admission.

To minimize the impact of acute infection on lymphocyte counts, we defined baseline lymphocyte as the earliest available result obtained 7–30 days before ICU admission. Pre-existing (baseline) lymphopenia was defined as baseline lymphocyte count < 1 × 10^9^/L, while sepsis-induced lymphopenia referred to peripheral blood lymphocyte count < 1 × 10^9^/L on the day of ICU admission in patients with normal baseline lymphocyte counts. In order to improve the accuracy of the conclusion, we changed the cut-off for lymphopenia to 0.8 × 10^9^/L and conducted sensitivity analysis.

Demographic characteristics, underlying diseases (including immunocompromised status), and infection sites were extracted from the electronic medical record system. Disease severity was assessed using Acute Physiology and Chronic Health Evaluation II (APACHE II) [15] score and Sequential Organ Failure Assessment (SOFA) [16] score recorded on ICU admission day. We also collected organ support measures, laboratory parameters, and clinical outcomes including ICU and total hospital length of stay, 30-day all-cause mortality post-ICU admission and in-hospital mortality.

Continuous variables were assessed for normality using Shapiro-Wilk test. Normally distributed data were presented as mean ±SD and analyzed using independent t-test (two groups) or one-way ANOVA (multiple groups). Non-normal data were expressed as median (interquartile range [IQR]) with Mann-Whitney U test (two groups) or Kruskal-Wallis test (multiple groups). Categorical variables were shown as n (%) and compared by chi-square test. Variables considered clinically important by the investigators, along with those with p-value < 0.1 in univariate analysis, were included in multivariable logistic regression. Collinearity was evaluated using variance inflation factor (VIF) and the model was considered acceptable if VIF < 10. Model fit was assessed by Hosmer-Lemeshow test (*p* > 0.05 indicating good fit). Odds ratios (OR) with 95% confidence interval (CI) were calculated. Survival analysis used Kaplan-Meier method with log-rank test. All analyses were performed using SPSS 22.0 and R 4.3.2, with *p* < 0.05 considered statistically significant.

## Results

### Baseline demographic and clinical characteristics

**Fig. 1:**
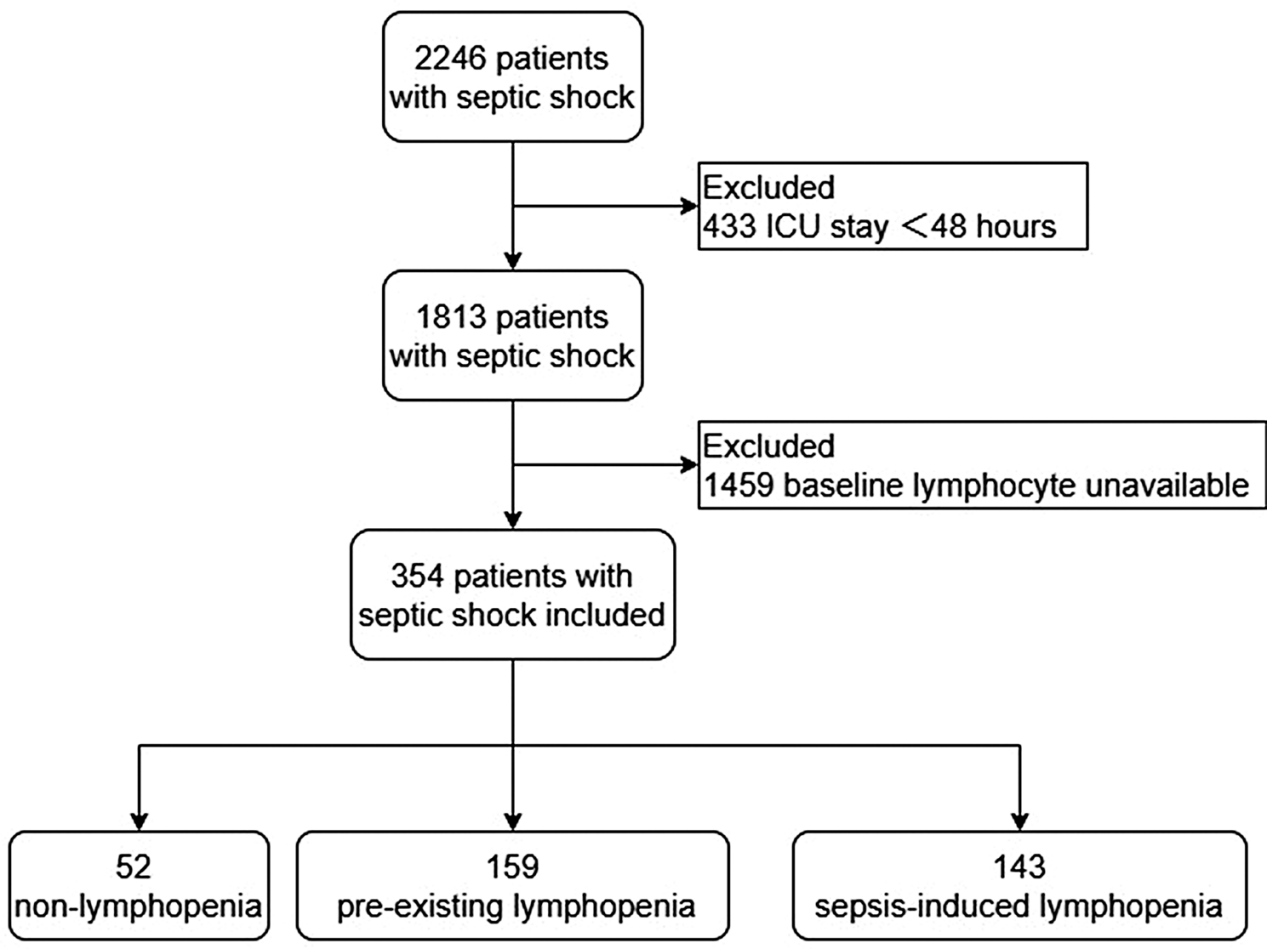
Flowchart of participants through this study

During the study period from January 2013 to June 2023, a total of 354 patients meeting the diagnostic criteria for septic shock with available lymphocyte count results within 7–30 days prior to ICU admission were enrolled (Fig. 1). The study population demonstrated a male predominance (63.5%) with a median age of 65 (IQR 54, 74) years. The temporal relationship between baseline lymphocyte assessment and ICU admission revealed a median interval of 16 days (IQR 11–26). The crude 30-day all-cause mortality reached 44.4% and in-hospital mortality was 60.5%.

### Comparisons of clinical characteristics stratified by lymphocyte profiles

The study population was systematically categorized into three distinct groups based on lymphocyte profiles: the non-lymphopenia group (*n* = 52), pre-existing lymphopenia group (*n* = 159), and sepsis-induced lymphopenia group (*n* = 143). Demographic parameters including age and gender distribution showed no statistically significant difference (*p* > 0.05).

Notably, immune status revealed that 69.8% of pre-existing lymphopenia patients had documented immunocompromised conditions at baseline. Comparative analysis of clinical parameters including primary infection sites, disease severity (APACHE II and SOFA scores), and requirements for organ supports showed no significant differences among the three groups (*p* > 0.05 for all comparisons). Mortality analysis demonstrated divergent outcomes, with the pre-existing lymphopenia group exhibiting the highest 30-day mortality (50.9%), while the mortality in sepsis-induced lymphopenia patients and non-lymphopenia groups were 37.1% and 44.2%, respectively (Table 1).

**Table 1:** Characteristics of non-lymphopenia patients with septic shock grouped by presence and timing of lymphopenia

	Non-lymphopenia （*n* = 52)	Pre-existing lymphopenia ^a^ （*n* = 159)	Sepsis-induced lymphopenia ^b^ （*n* = 143）	P value
Age (years)	70 (60, 80)	65 (54, 74)	63 (53, 73)	0.071
Male	40 (76.9)	101 (63.5)	84 (58.7)	0.066
Hospital stay before ICU admission (days)	17 (11, 32)	15 (10, 25)	11 (5, 22)	< 0.001
Baseline lymphocyte ^c^ (×10^9^/L)	1.48 (1.23, 1.89)	0.63 (0.44, 0.79)	1.48 (1.20, 2.04)	< 0.001
Baseline-ICU interval ^d^ (days)	17 (11, 26)	16 (11, 26)	17 (11, 25)	0.835
**Comorbidities**				
Hypertension	20 (38.5)	55 (34.6)	44 (30.8)	0.567
Diabetes mellitus	19 (36.5)	30 (18.9)	35 (24.5)	0.033
COPD	1 (1.9)	1 (0.6)	0 (0.0)	0.282
Congestive heart failure	3 (5.8)	3 (1.9)	7 (4.9)	0.261
Liver cirrhosis	1 (1.9)	4 (2.5)	2 (1.4)	0.784
Immunocompromised status	18 (34.6)	111 (69.8)	79 (55.2)	< 0.001
Hematological malignancy	3 (5.8)	21 (13.2)	17 (11.9)	0.343
Autoimmune Disease	3 (5.8)	28 (17.6)	13 (9.1)	0.023
Solid tumor	9 (17.3)	41 (25.8)	42 (29.4)	0.236
**Disease severity at ICU admission**				
APACHE (points)	23 (17, 29)	23 (19, 28)	21 (17, 26)	0.123
SOFA (points)	10 (8, 11)	11 (9, 13)	10 (8, 12)	0.085
**Sites of infection**				
Bloodstream	6 (11.5)	26 (16.5)	22 (15.4)	0.694
Pulmonary	28 (53.8)	92 (57.9)	63 (44.1)	0.053
Intra-abdominal	16 (30.8)	40 (25.3)	54 (37.8)	0.066
Urinary tract	0 (0.0)	5 (3.2)	7 (4.9)	0.243
Skin and soft tissue	2 (3.8)	1 (0.6)	4 (2.8)	0.235
**Organ support at ICU admission**				
PaO_2_/FiO_2_ (mmHg)	297 (226, 380)	244 (165, 352)	272 (183, 371)	0.269
Invasive mechanical ventilation	34 (65.4)	110 (69.2)	99 (69.2)	0.860
CRRT	8 (15.4)	44 (27.7)	28 (19.6)	0.099
NE (μg/kg/min)	0.50 (0.18, 1.00)	0.53 (0.20, 1.13)	0.50 (0.20, 1.00)	0.676
NE > 1 μg/kg/min	6 (11.5)	18 (11.3)	23 (16.1)	0.440
**Laboratory Results at ICU admission**				
Lactate (mmol/L)	2.2 (1.4, 4.0)	2.4 (1.5, 4.2)	2.7 (1.7, 4.3)	0.203
Neutrophils (×10^9^/L)	9.87 (8.07, 17.16)	8.22 (4.42, 14.69)	7.37 (4.44, 11.82)	0.001
Lymphocyte (×10^9^/L)	1.48 (1.18, 1.90)	0.46 (0.26, 0.69)	0.49 (0.32, 0.73)	< 0.001
Platelet (×10^9^/L)	164 (100, 266)	98 (50, 170)	132 (77, 189)	< 0.001
Hemoglobin (g/L)	94 (84, 110)	82 (73, 97)	93 (80, 109)	< 0.001
Albumin (g/L)	29 (26, 32)	28 (25, 31)	29 (26, 32)	0.164
Total bilirubin (μmol/L)	15.1 (11.3, 22.7)	17.5 (11.1, 33.6)	17.4 (11.8, 31.0)	0.364
Creatinine (μmol/L)	91 (63, 146)	93 (59, 160)	97 (67, 144)	0.980
Procalcitonin (ng/ml)	3.58 (0.48, 5.99)	2.95 (0.66, 10.17)	6.35 (1.75, 26.75)	0.003
**Outcomes**				
30-day mortality ^e^	23 (44.2)	81 (50.9)	53 (37.1)	0.053
Hospital mortality	28 (53.8)	110 (69.2)	76 (53.1)	0.010
Length of stay (days)	16 (10, 28)	14 (8, 33)	16 (8, 38)	0.919
Length of ICU stay (days)	8 (4, 13)	8 (5, 15)	6 (4, 11)	0.015

Data are presented as n (%) or median (IQR). APACHE, Acute Physiology and Chronic Health evaluation; COPD, Chronic obstructive pulmonary disease; CRRT, continuous renal replacement therapy; ICU, intensive care unit; NE, Norepinephrine; SOFA, Sequential Organ Failure Assessment

^a^ Pre-existing lymphopenia referred to baseline lymphocyte count less than 1 × 10^9^/L. Baseline lymphocyte referred to the earliest lymphocyte count taken from the blood routine results within 7–30 days before ICU admission

^b^ Sepsis-induced lymphopenia indicated that the lymphocyte count taken from the blood routine results on the day of ICU admission was less than 1 × 10^9^/L without baseline lymphopenia

^c^ Baseline lymphocyte referred to the earliest lymphocyte count taken from the blood routine results within 7–30 days before ICU admission

^d^ The interval between the date of baseline lymphocyte measurement and the date of ICU admission

^e^ From the day of ICU admission

**Fig. 2:**
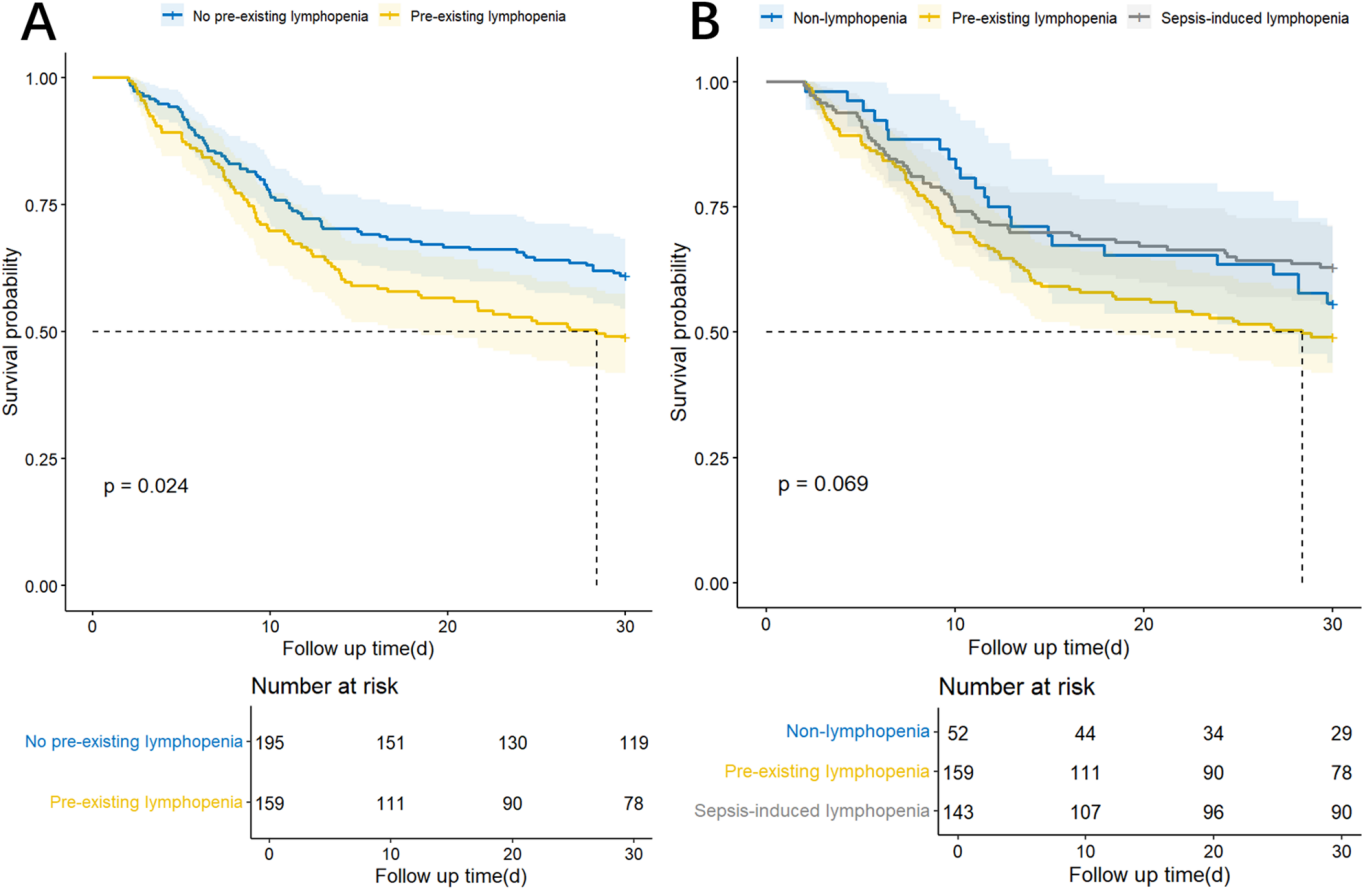
Kaplan–Meier survival curves of patients with septic shock patients according to the presence and timing of lymphopenia. (**A**) comparison between patients without pre-existing lymphopenia and those with pre-existing lymphopenia (log-rank *p* = 0.024); (**B**) three-group survival comparison among non-lymphopenia, pre-existing lymphopenia, and sepsis-induced lymphopenia using the log-rank test (overall *p* = 0.069). Pairwise log-rank comparisons revealed that there was no significant difference in 30-day mortality between patients in the pre-existing lymphopenia group and those in the sepsis-induced lymphopenia group or the non-lymphopenia group (*p* values were 0.075 and 0.397, respectively). Additionally, no significant difference was found between the sepsis-induced lymphopenia group and the non-lymphopenia group (*p* = 0.579)

Survival analysis using Kaplan-Meier curve revealed significantly inferior outcomes in patients with pre-existing lymphopenia compared to those with no pre-existing lymphopenia (*p* = 0.024). However, the three-group comparison incorporating pre-existing lymphopenia, sepsis-induced lymphopenia, and non-lymphopenia groups failed to demonstrate statistically significant survival differences (*p* = 0.069). Pairwise log-rank comparisons revealed that there was no significant difference in 30-day mortality between patients in the pre-existing lymphopenia group and those in the sepsis-induced lymphopenia group or the non-lymphopenia group (*p* values were 0.075 and 0.397, respectively). Additionally, no significant difference was found between the sepsis-induced lymphopenia group and the non-lymphopenia group (*p* = 0.579), as shown in Fig. 2.

### Multivariable analysis of risk factors for 30-day mortality

As delineated in Table 2, univariate analysis between survivors and non-survivors revealed that decedents exhibited significantly greater disease severity and higher requirements for advanced organ support modalities. To elucidate the independent prognostic value of lymphocyte patterns, we constructed three distinct multivariable logistic regression models (Table 3).

**Table 2:** Univariate analysis of risk factors associated with 30-day mortality in patients with septic shock

	Total （*n* = 354)	Survivors（*n* = 197)	Non-survivors （*n* = 157）	P value
Age (years)	65 (54, 74)	67 (55, 75)	64 (53, 72)	0.059
Male	225 (63.5)	63 (71.6)	72 (60.5)	0.465
Hospital stay before ICU admission (days)	14 (8, 25)	13 (8, 25)	14 (9, 25)	0.778
Baseline lymphocyte ^a^ (×109/L)	1.06 (0.68, 1.50)	1.12 (0.73, 1.53)	0.98 (0.60, 1.40)	0.011
Baseline lymphopenia ^b^	159 (44.9)	78 (39.6)	81 (51.6)	0.032
Baseline- ICU interval ^c^ (days)	16.5 (11, 26)	16 (11, 26)	17 (11, 26)	0.644
**Comorbidities**				
Hypertension	119 (33.6)	69 (35.0)	50 (31.8)	0.606
Diabetes mellitus	84 (23.7)	46 (23.4)	38 (24.2)	0.951
COPD	2 (0.6)	0 (0.0)	2 (1.3)	0.382
Congestive heart failure	13 (3.7)	6 (3.0)	7 (4.5)	0.676
Liver cirrhosis	7 (2.0)	3 (1.5)	4 (2.5)	0.761
Immunocompromised	208 (58.8)	115 (58.4)	93 (59.2)	0.956
Hematological malignancy	41 (11.6)	23 (11.7)	18 (11.5)	1.000
Autoimmune Disease	44 (12.4)	23 (11.7)	21 (13.4)	0.749
Solid tumor	92 (26.0)	51 (25.9)	41 (26.1)	1.000
**Disease severity at ICU admission**				
APACHE (points)	22 (18, 27)	21 (17, 25)	24 (19, 30)	< 0.001
SOFA (points)	10 (8, 13)	10 (8, 12)	11 (9, 14)	< 0.001
**Sites of infection**				
Bloodstream	54 (15.3)	28 (14.2)	26 (16.7)	0.626
Pulmonary	183 (51.7)	96 (48.7)	87 (55.4)	0.253
Intra-abdominal	110 (31.2)	73 (37.1)	37 (23.7)	0.010
Urinary tract	12 (3.4)	11 (5.6)	1 (0.6)	0.024
**Organ support at ICU admission**				
PaO_2_/FiO_2_ (mmHg)	265 (175, 367)	285 (187, 386)	252 (157, 333)	0.020
Invasive mechanical ventilation	243 (68.6)	124 (62.9)	119 (75.8)	0.013
CRRT	80 (22.6)	33 (16.8)	47 (29.9)	0.005
NE (μg/kg/min)	0.50 (0.20, 1.00)	0.41 (0.20, 1.00)	0.80 (0.40, 2.00)	< 0.001
**Laboratory Results at ICU admission**				
Lactate (mmol/L)	2.5 (1.6, 4.3)	2.3 (1.5, 3.7)	2.8 (1.8, 5.2)	0.002
lactate > 4 mmol/L	93 (26.3)	38 (19.3)	55 (35.0)	0.001
Neutrophils (×10^9^/L)	8.37 (4.81, 13.92)	8.64 (5.32, 13.86)	7.85 (3.84, 14.31)	0.098
Lymphocyte (×10^9^/L)	0.54 (0.32, 0.86)	0.61 (0.39, 0.92)	0.42 (0.22, 0.78)	< 0.001
Lymphopenia ^d^	287 (81.1)	158 (80.2)	129 (82.2)	0.740
Platelet (×10^9^/L)	124 (69, 186)	136 (92, 196)	90 (48, 168)	< 0.001
Hemoglobin (g/L)	89 (77, 106)	91.00 (81, 110)	84 (72, 102)	0.002
Albumin (g/L)	29 (25, 32)	29 (26, 32)	28 (25, 31)	0.009
Total bilirubin (μmol/L)	17.2 (11.5, 31.2)	15.5 (11.1, 27.9)	19.7 (12.4, 41.5)	0.013
Creatinine (μmol/L)	95 (63, 151)	93 (62, 148)	97 (64, 152)	0.799
Procalcitonin (ng/ml)	4.11 (0.78, 15.87)	3.50 (0.73, 13.13)	4.40 (1.22, 22.00)	0.262

Data are presented as n (%) or median (IQR). APACHE, Acute Physiology and Chronic Health evaluation; COPD, Chronic obstructive pulmonary disease; CRRT, continuous renal replacement therapy; ICU, intensive care unit; NE, Norepinephrine; SOFA, Sequential Organ Failure Assessment

^a^ Baseline lymphocyte referred to the earliest lymphocyte count taken from the blood routine results within 7–30 days before ICU admission

^b^ Baseline lymphocyte < 1 × 10^9^/L

^c^ The interval between the date of baseline lymphocyte measurement and the date of ICU admission

^d^ Lymphopenia indicated that the lymphocyte count taken from the blood routine results on the day of ICU admission was less than 1 × 10^9^/L

**Table 3:** Multivariable logistic regression analysis of risk factors associated with 30-day mortality in patients with septic shock

	Risk factors	OR (*95%*CI)	P
Model 1	Baseline lymphopenia ^a^	3.67 (1.61, 8.79)	0.003
	Norepinephrine (μg/kg/min)	2.07 (1.37, 3.37)	0.001
	Lactate > 4 mmol/L	2.70 (1.13, 6.69)	0.028
	Lymphopenia at ICU admission ^b^	0.95 (0.28, 3.37)	0.928
Model 2	Baseline lymphocyte ^a^	0.54 (0.31, 0.88)	0.018
	Norepinephrine (μg/kg/min)	2.07 (1.38, 3.33)	0.001
	Lactate > 4 mmol/L	2.76 (1.15, 6.90)	0.026
	Lymphocyte at ICU admission	0.98 (0.50, 1.76)	0.948
Model 3	Norepinephrine (μg/kg/min)	2.03 (1.30, 3.18)	0.002
	Lactate > 4 mmol/L	2.51 (1.02, 6.16)	0.044
	Lymphocyte types		0.064
	Non-lymphopenia	Reference	
	Pre-existing lymphopenia ^a^	2.82 (0.78, 10.29)	0.115
	Sepsis-induced lymphopenia ^c^	0.70 (0.19, 2.52)	0.582

CI: 95% confidence interval. OR, odds ratio

Variables in **model 1** included age, male, baseline lymphopenia, intra-abdominal infection, APACHE, SOFA, Norepinephrine (μg/kg/min), PaO_2_/FiO_2_, continuous renal replacement therapy, lactate > 4 mmol/L, lymphopenia at ICU admission, platelet count, hemoglobin, albumin, total bilirubin. The *p* value for the Hosmer-Lemeshow test was 0.746 and the VIF values of all variables were less than 3

**Model 2** analyzed baseline lymphocyte and lymphocyte at ICU admission as continuous variables, while the remaining variables are the same as those in **Model 1**. The *p* value for the Hosmer-Lemeshow test was 0.804 and the VIF values of all variables were less than 2

**Model 3** categorized patients into three groups of lymphocyte types (non-lymphopenia, pre-existing lymphopenia and sepsis-induced lymphopenia) based on baseline lymphocytes and lymphocytes at ICU admission, with other variables being the same as those in Model 1. The *p* value for the Hosmer-Lemeshow test was 0.702 and the VIF values of all variables were less than 3

^a^ Baseline lymphopenia and pre-existing lymphopenia both referred to baseline lymphocyte count less than 1 × 10^9^/L. Baseline lymphocyte referred to the earliest lymphocyte count taken from the blood routine results within 7–30 days before ICU admission

^b^ Lymphopenia at ICU admission indicated that the lymphocyte count taken from the blood routine results on the day of ICU admission was less than 1 × 10^9^/L

^c^ Sepsis-induced lymphopenia indicated that the lymphocyte count taken from the blood routine results on the day of ICU admission was less than 1 × 10^9^/L without baseline lymphopenia

Model 1 incorporated baseline lymphopenia and lymphopenia at ICU admission as separate covariates in the regression analysis. This model identified baseline lymphopenia as an independent predictor of 30-day mortality (OR 3.67, 95% CI 1.61–8.79), while lymphopenia at ICU admission showed no significant association with mortality (OR 0.95, 95% CI 0.28–3.37). Model 2 analyzed baseline lymphocyte and lymphocyte at ICU admission as continuous variables and the results was consistent with Model 1. Model 3 alternatively classified lymphocyte status as a three-level categorical variable (pre-existing lymphopenia, sepsis-induced lymphopenia, and non-lymphopenia). In this configuration, neither pre-existing lymphopenia nor sepsis-induced lymphopenia demonstrated statistically significant mortality prediction compared to the non-lymphopenia reference group.

All the regression models consistently identified two robust independent predictors of mortality: the norepinephrine dose (*p* < 0.01 for all models) and the presence of arterial lactate elevation exceeding 4 mmol/L at ICU admission (*p* < 0.05 for all models). The models demonstrated satisfactory goodness-of-fit as evidenced by non-significant Hosmer-Lemeshow test results (*p* > 0.05 in all models), and VIF values for all included covariates remained below the prespecified threshold of 10, indicating acceptable multicollinearity levels (Table 3).

To ensure the robustness of our findings, we conducted additional sensitivity analyses. First, as the conventional lymphopenia threshold ( < 1.0 × 10^9^/L) varies among previous studies, we repeated the analyses using an alternative cut-off of 0.8 × 10^9^/L. The results were consistent with the main analysis: pre-existing lymphopenia remained an independent predictor of 30‑day mortality (OR 3.44, 95% CI 1.42–8.69), whereas lymphopenia at ICU admission was not (OR 1.39, 95% CI 0.56–3.61) (Supplementary Table 1). Second, treatment strategies may have evolved over time because this study included patients with septic shock over a 10‑year period. To address this concern, we performed an additional sensitivity analysis restricted to patients admitted after 2017 (*n* = 222, 43.2% mortality), when the Sepsis‑3 criteria had been widely adopted. The results remained consistent with the main analysis (Supplementary Table 2).

## Discussion

To evaluate the influence of baseline lymphocyte counts on 30-day mortality of patients with septic shock, this study introduced the novel concept of baseline lymphocytes and systematically compared clinical characteristics and outcomes among patients with pre-existing lymphopenia (baseline lymphopenia), sepsis-induced lymphopenia, and non-lymphopenia. Our findings demonstrated that, among septic-shock patients, those with pre-existing lymphopenia exhibited significantly higher 30-day mortality than those with no pre-existing lymphopenia, whereas patients with sepsis-induced lymphopenia on ICU admission had comparable mortality rates to non-lymphopenia individuals. Multivariable analysis confirmed pre-existing lymphopenia as an independent predictor of mortality, while sepsis-induced lymphopenia at ICU admission was not associated with increased risk.

A growing body of evidence has identified lymphopenia as a pivotal factor in sepsis pathophysiology and prognosis. Sepsis-induced lymphopenia, typically defined as an absolute lymphocyte count < 1.0 × 10^9^/L, has been consistently linked to increased risks of secondary infections, multiple organ failure, and mortality [17–19]. Mechanistically, this association reflect two major disruptions in lymphocyte homeostasis during sepsis: excessive apoptosis mediated by Fas/FasL signaling, mitochondrial dysfunction, and endoplasmic reticulum stress, and impaired lymphocyte proliferation due to reduced thymic output, bone marrow exhaustion, and upregulated immune checkpoints such as PD-1/PD-L1 and CTLA-4) [17, 20, 21]. These processes collectively deplete lymphocyte populations, particularly T, B, and NK cells) and induce immunosuppression, creating a “lymphopenia-immunosuppression-adverse outcome” cycle [17].

Previous studies have focused on post-infection lymphocyte dynamics (i.e., lymphocyte counts at or after sepsis diagnosis), while research on lymphocytes before the onset of sepsis remains extremely limited. A large-scale prospective study followed a community population (*n* = 98344) for an average of 6 years and identified 2,352 subjects with lymphopenia and demonstrated, after age and sex adjustment, that lymphopenia was associated with increased risks of sepsis development (HR 1.51) and infection-related mortality (HR 1.7) [13]. Similarly, preoperative lymphocyte counts were found to be significantly correlated with postoperative sepsis risk in gastrointestinal surgery patients [22]. Lymphocytes were recognized as critical regulators of immune homeostasis in critical illness, particularly sepsis [23, 24]. An intact lymphocyte repertoire is essential for effective pathogen clearance [25].

This study is the first to explore the relationship between baseline lymphocytes and clinical outcomes in patients with septic shock. Our findings suggest that pre-existing lymphopenia may be a more critical determinant of patient outcomes than sepsis-induced lymphopenia. We propose that sepsis-induced lymphopenia may reflect a transient, reversible redistribution induced by sepsis, whereas pre-existing lymphopenia signifies a state of pre-existing immune exhaustion or clonal depletion, leading to worse outcomes. Theoretically, pre-infection lymphocyte counts directly influence the body’s initial immune reserve. Individuals with sufficient baseline lymphocytes may possess a superior capacity for antigen recognition and clearance, thereby mitigating early immune dysregulation. Conversely, those with low baseline levels may rapidly develop severe immunosuppression due to an inadequate immune reserve, accelerating disease progression and ultimately leading to poor outcomes.

Interestingly, baseline lymphopenia remained an independent predictor of 30-day mortality in the multivariable logistic regression analysis when entered as a binary variable (Models 1–2), but this association was no longer statistically significant when lymphocyte status was categorized into three groups: pre-existing lymphopenia, sepsis-induced lymphopenia on ICU admission, and non-lymphopenia (Model 3). The differences observed between the models may be explained by several factors. First, dividing the cohort into three subgroups inevitably reduced the sample size and statistical power, leading to wider confidence intervals. Second, the subgroup with sepsis-induced lymphopenia on ICU admission represents a biologically heterogeneous population, as many patients may experience only transient lymphocyte redistribution rather than sustained immune depletion. Third, from an immunological perspective, baseline lymphopenia reflects a state of chronic immune exhaustion and limited immune reserve, whereas sepsis-induced(acute) lymphopenia may correspond to a reversible, stress‑related phenomenon. Therefore, rather than indicating contradictory findings, the difference between models underscores the complex and dynamic nature of immune alterations during sepsis progression.

As the primary objective of our study was to investigate the impact of pre‑existing lymphopenia on clinical outcomes in patients with septic shock, it was essential to include only those with available pre‑admission laboratory data, allowing us to assess baseline lymphocyte levels. However, this approach may have resulted in systematic differences between patients included in the study and those excluded. For instance, individuals with more frequent pre‑admission testing may have underlying chronic diseases or better access to healthcare, which could influence both baseline immune status and clinical outcomes, thereby affecting the generalizability of our findings.

This study also has some limitations that should be acknowledged. First, our study is a single center study, however, the relatively large sample size may enhance its generalizability. In addition, the definition of baseline lymphocytes as the earliest available value within 7–30 days before ICU admission involves a certain degree of subjectivity. However, it was a practical compromise—lymphocyte values obtained within one week before ICU admission are often influenced by ongoing infection, whereas those collected more than 30 days earlier may be affected by other, unrelated infections. Although this definition may not perfectly distinguish between chronic and transient lymphopenia, it provides a clinically reasonable and practically applicable approximation that allows consistent comparison across patients. Besides, due to incomplete clinical documentation, our analysis was restricted to disease severity and organ support parameters, without key variables such as pathogens, antibiotic regimens, or fluid resuscitation strategies. The absence of detailed pathogen information may have limited our ability to explore potential interactions among baseline lymphocyte status, pathogen type, and clinical outcomes, which could provide important insights into host–pathogen immune dynamics. Furthermore, the lack of lymphocyte subset analysis (CD4^+^/CD8^+^) represents a major biological limitation, preventing a more mechanistic understanding of how specific immune cell populations contribute to sepsis outcomes. Future prospective studies incorporating microbiological profiles and detailed immunophenotyping are warranted to achieve a more comprehensive evaluation of host immune responses across different infection contexts.

## Conclusions

In this comprehensive analysis of septic shock patients with available pre-admission lymphocyte data, we demonstrate that pre-existing lymphopenia (baseline) lymphopenia, but not lymphopenia at ICU admission (sepsis-induced lymphopenia), served as an independent predictor of 30-day mortality. This finding highlights the prognostic importance of pre-existing immune status in outcomes of patients with septic shock. These results suggest that historical lymphocyte count level should be incorporated into risk stratification paradigms for patients with septic shock.

## Electronic supplementary material

Below is the link to the electronic supplementary material.

Supplementary Material 1

## Data Availability

The datasets generated and/or analyzed during the current study are available from the corresponding author on reasonable request.

## Abbreviations

APACHE: Acute Physiology and Chronic Health evaluation
CI: Confidence interval
COPD: Chronic obstructive pulmonary disease
CRRT: Continuous renal replacement therapy
ICU: Intensive care unit
IQR: Interquartile range
OR: Odds ratio
SOFA: Sequential Organ Failure Assessment
VIF: Variance inflation factor
