# Supplementary material for: Impact of baseline lymphopenia on prognosis of patients with septic shock
Source: BMC Infect Dis. 2025 Dec 15;25:1720. doi: 10.1186/s12879-025-12078-9 (PMC12706955; doi:10.1186/s12879-025-12078-9)
Supplement: Supplementary file 1 — Supplementary Material 1 [file 12879_2025_12078_MOESM1_ESM.docx]

Supplementary Table 1. Multivariable logistic regression analysis of factors associated with 30-day mortality in patients with septic shock.

|  | Risk factors | OR *(95%*CI*)* | *P* |
| --- | --- | --- | --- |
| Model 1 | Baseline lymphopenia ^a^ | 3.44 (1.42, 8.69) | 0.007 |
|  | Norepinephrine (μg/kg/min) | 2.02(1.34, 3.29) | 0.002 |
|  | Lactate >4mmol/L | 2.63 (1.10, 6.47) | 0.031 |
|  | Lymphopenia at ICU admission ^b^ | 1.39 (0.56, 3.61) | 0.487 |
| Model 2 | Norepinephrine (μg/kg/min) | 2.01 (1.33, 3.26) | 0.002 |
|  | Lymphocyte types |  |  |
|  | Non-lymphopenia | Reference |  |
|  | Pre-existing lymphopenia ^a^ | 4.93 (1.55, 16.88) | 0.008 |
|  | Sepsis-induced lymphopenia ^c^ | 1.58 (0.58, 4.63) | 0.384 |

APACHE, Acute Physiology and Chronic Health evaluation; SOFA, Sequential Organ Failure Assessment; CI: 95% confidence interval. OR, odds ratio.

Variables in **model 1** included age, male, baseline lymphopenia, intra-abdominal infection, APACHE Ⅱ, SOFA, Norepinephrine (μg/kg/min), PaO_2_/FiO_2_, continuous renal replacement therapy, lactate >4mmol/L, lymphopenia at ICU admission, platelet count, hemoglobin, albumin, total bilirubin. The P value for the Hosmer-Lemeshow test was 0.727 and the VIF values of all variables were less than 3.

**Model 2** categorized patients into three groups of lymphocyte types (non-lymphopenia, pre-existing lymphopenia and sepsis-induced lymphopenia) based on baseline lymphocytes and lymphocytes at ICU admission, with other variables being the same as those in Model 1. The P value for the Hosmer-Lemeshow test was 0.549 and the VIF values of all variables were less than 3.

^a^ Baseline lymphopenia and pre-existing lymphopenia both referred to baseline lymphocyte count less than 0.8×10^9^/L. Baseline lymphocyte referred to the earliest available result obtained 7-30 days before ICU admission.

^b^ Lymphopenia at ICU admission indicated that the lymphocyte of count taken from the blood routine results on the day of ICU admission was less than 0.8×10^9^/L.

^c^ Sepsis-induced lymphopenia indicated that the lymphocyte of count taken from the blood routine results on the day of ICU admission was less than 0.8×10^9^/L without baseline lymphopenia.

Supplementary Table 2. Multivariable logistic regression analysis of factors associated with 30-day mortality in patients with septic shock (only included patients after 2017).

| Risk factors | OR *(95%*CI*)* | *P* |
| --- | --- | --- |
| Baseline lymphopenia ^a^ | 3.73 (1.35, 11.02) | 0.013 |
| Norepinephrine (μg/kg/min) | 2.40 (1.22, 5.37) | 0.020 |
| Mechanical ventilation | 4.31 (1.16, 18.85) | 0.038 |
| Lymphopenia at ICU admission ^b^ | 2.93 (0.67, 16.50) | 0.178 |

APACHE, Acute Physiology and Chronic Health evaluation; SOFA, Sequential Organ Failure Assessment; CI: 95% confidence interval. OR, odds ratio.

A total of 222 patients were included, among whom 96 died. Variables in this model included age, male, baseline lymphopenia, APACHE Ⅱ, SOFA, Norepinephrine (μg/kg/min), mechanical ventilation, continuous renal replacement therapy, lactate >4mmol/L, lymphopenia at ICU admission, platelet count, hemoglobin, albumin, total bilirubin. The P value for the Hosmer-Lemeshow test was 0.870 and the VIF values of all variables were less than 2.

^a^ Baseline lymphopenia referred to baseline lymphocyte count less than 1×10^9^/L. Baseline lymphocyte referred to the earliest available result obtained 7-30 days before ICU admission.

^b^ Lymphopenia at ICU admission indicated that the lymphocyte of count taken from the blood routine results on the day of ICU admission was less than 1×10^9^/L.
